# Supplementary material for: Cholesterol as an Endogenous Ligand of ERRα Promotes ERRα-Mediated Cellular Proliferation and Metabolic Target Gene Expression in Breast Cancer Cells
Source: Cells. 2020 Jul 23;9(8):1765. doi: 10.3390/cells9081765 (PMC7463712; doi:10.3390/cells9081765)
Supplement: Supplementary file 1 [file cells-09-01765-s001.pdf]

Table S1. The primer sequences used for qPCR

Target Gene		Sequence: 5' to 3'
SOD2	F	CTGGACAAACCTCAGCCCTAAC
	R	AACCTGAGCCTTGGACACCAAC
IDH3A	F	TCGGTGTGACACCAAGTGGCAA
	R	TTCGCCATGTCCTTGCCTGCAA
VEGF	F	TTGCCTTGCTGCTCTACCTCCA
	R	GATGGCAGTAGCTGCGCTGATA
PDK4	F	AGGTGGAGCATTTCTCGCGCTA
	R	GAATGTTGGCGAGTCTCACAGG
SPP1	F	CGAGGTGATAGTGTGGTTTATGG
	R	GCACCATTCAACTCCTCGCTTTC
GSTM1	F	TGATGTCCTTGACCTCCACCGT
	R	GCTGGACTTCATGTAGGCAGAG
ERR1	F	CCACTATGGTGTGGCATCCTGT
	R	GGTGATCTCACACTCGTTGGAG
PGC-1	F	CCAAAGGATGCGCTCTCGTTCA
	R	CGGTGTCTGTAGTGGCTTGACT
GAPDH	F	GTCTCCTCTGACTTCAACAGCG
	R	ACCACCCTGTTGCTGTAGCCAA
